# A multi-constraint and multi-objective optimization layout method for a mine water inrush monitoring network

**DOI:** 10.1038/s41598-023-39118-1

**Published:** 2023-07-21

**Authors:** Zhili Du, Qiang Wu, Yingwang Zhao, Xiaoyan Zhang, Yi Yao

**Affiliations:** 1 College of Geoscience and Surveying Engineering, China University of Mining and Technology (Beijing), Beijing, China; 2 National Engineering Research Center of Coal Mine Water Hazard Controlling, Beijing, China

**Keywords:** Hydrology, Energy science and technology

## Abstract

Mine water inrush can cause property losses and casualties, but current theoretical and technological approaches cannot accurately predict such events. Through the networked deployment of water level sensors along a mine roadway, a mine water inrush monitoring network was developed, and a multi-constraint and multi-objective optimal deployment method was established. By setting practical constraints of the mining area, water inrush risk level, and installation at specified locations, and considering two objective functions of minimum total cost and minimum average monitoring time, a mathematical model was established. The non-dominated sorting genetic algorithm II (NSGA-II) was designed to solve the model. The method temporally and spatially optimized the network, which was then verified in the Beiyangzhuang coal mine in north China. The average response time of the monitoring network was 916 s using only 28 water level sensors. The higher the water inrush risk level, the shorter the monitoring network response time. Under the 2, 3, and 4 risk levels, the network’s response time to simulated water inrush accidents was less than 3000, 2100, and 900 s, respectively. The multi-constraint and multi-objective optimization layout method further enhanced the effectiveness of the network, providing a novel system for the early warning of mine water inrush.

## Introduction

China is a major coal producing country, accounting for almost half of the world’s total coal production. About 90% of China’s coal production comes from underground mines^1,2^. With the increase in mining depth and mining scale, the geological structures of underground mines are becoming increasingly complex, and mining engineering problems are therefore becoming more common. Approximately 60% of mine accidents are related to groundwater^3,4^. Mine water inrush is one of the main problems threatening coal mine safety^5^ and has therefore been the subject of several studies in recent years. Ruan et al. established an integrated decision-making model for water inrush based on a comprehensive analysis of the factors controlling the mine water inrush risk and an improvement of the analytic hierarchy process (AHP), which enhanced the accuracy of water inrush probability prediction^6^. Shi et al. established a water inrush risk index (WIRI) model to effectively predict the water inrush risk of the Ordovician aquifer in the Xinwen coalfield, China^7^. Zhang et al. proposed a variable weight model to achieve the risk assessment of water inrush from the floor in the Taoyang Coal Mine^8^. He et al. proposed a bed-separation water inrush (BSWI) method to assess the risk of water inrush from the bottom layer of the Cretaceous strata in the Ordos Basin^9^. Wang et al. proposed a hazard identification method based on a fault tree analysis, which improved the risk control and prevention of mine water inrush accidents^10^. Zhang et al. developed an integrated floor water inrush model to predict the risk of water inrush caused by highly confined aquifers under complex stress conditions^11^. Ye et al. proposed a neural network prediction method based on deep learning and used the improved synthetic minority oversampling technique (ISMOTE) to improve the accuracy of water inrush predictions^12^. Studies of the prediction and risk assessment theory for mine water hazards have led to a decreasing trend of water inrush accidents over time^13^. However, disastrous water inrush accidents still occur periodically, causing huge property losses and even casualties^14^. Therefore, the monitoring and establishment of early warning systems for mine water inrush has become a research hotspot.

Zhou et al. used distributed fiber optic sensing technology (Brillouin optical time-domain reflectometer: BOTDR) and a 3D resistivity survey to dynamically monitor the characteristics of deformation and failure of a coal seam floor to prevent water inrush in a mine^15^. Ma et al. used a microseismic monitoring system to detect the progressive failure of a coal seam floor during mining in real-time to prevent water inrush from the floor^16^. Zhang et al. proposed a method for monitoring water inrush in coal mines using a distributed fuzzy clustering analysis of time-lapse electrical resistivity tomography^17^. Yang et al. used the constrained time-lapse resistivity inversion method to dynamically monitor damage to the floor during coal seam mining, thereby achieving predictions of water inrush in the working face^18^. Zhao et al. used real-time monitoring of temperature changes in the collapse columns in coal mines to predict the occurrence of water inrush^19^. Gao et al. used a novel thermal infrared analysis method to monitor rock fracture dynamics for the early warning of mine water inrush^20^. The monitoring and early warning of mine water inrush are currently achieved by monitoring the changes in a specific indicator. The location of monitoring points is mainly based on subjective experience and lacks theoretical support. Wu et al. successfully attempted to optimize the location of monitoring points using the set covering model based on facility location theory^21^. However, because they only considered cost as a goal, the results were not satisfactory. Multi-objective optimization can solve this problem and has been successfully applied in various engineering fields. Guo et al. applied multi-objective optimization methods to the field of cloud computing and optimized multi-objective task scheduling^22^. Azizi et al. applied multi-objective optimization methods to a novel poly-generation system based on a solar power tower for power generation, optimizing the exergetic efficiency of the system^23^. Nedaei et al. applied multi-objective optimization methods to an innovative multigeneration system based on a heliostat solar field to obtain the optimum operating conditions and achieve a desirable performance level^24^. Chai et al. applied multi-objective optimization methods to the field of wireless channels, achieving the optimization of spectrum allocation and power control in vehicle communication^25^.

This study developed a multi-constraint and multi-objective optimal deployment method for a mine water inrush monitoring network, which optimized the monitoring network in the spatial and temporal dimensions, thus reducing the network cost and improving its response time. The method further enriched the theoretical and practical aspects of monitoring, providing an early warning of mine water inrush.

The main outcomes of this study were as follows.

1. A water inrush monitoring network was proposed, and two objective functions were constructed: minimum total cost and minimum average monitoring time. The monitoring network was then temporally and spatially optimized.
2. Three main constraints (mining area, water inrush risk level, and installation at specified locations) that influenced the effectiveness of sensor deployment were extracted and quantified, and then a multi-constraint and multi-objective optimization model was established.
3. A multi-objective genetic algorithm (the non-dominated sorting genetic algorithm II: NSGA-II) was designed using the Python programming language to solve the multi-constraint and multi-objective optimization model.

## Methodology

### Problem description

**Figure 1:**
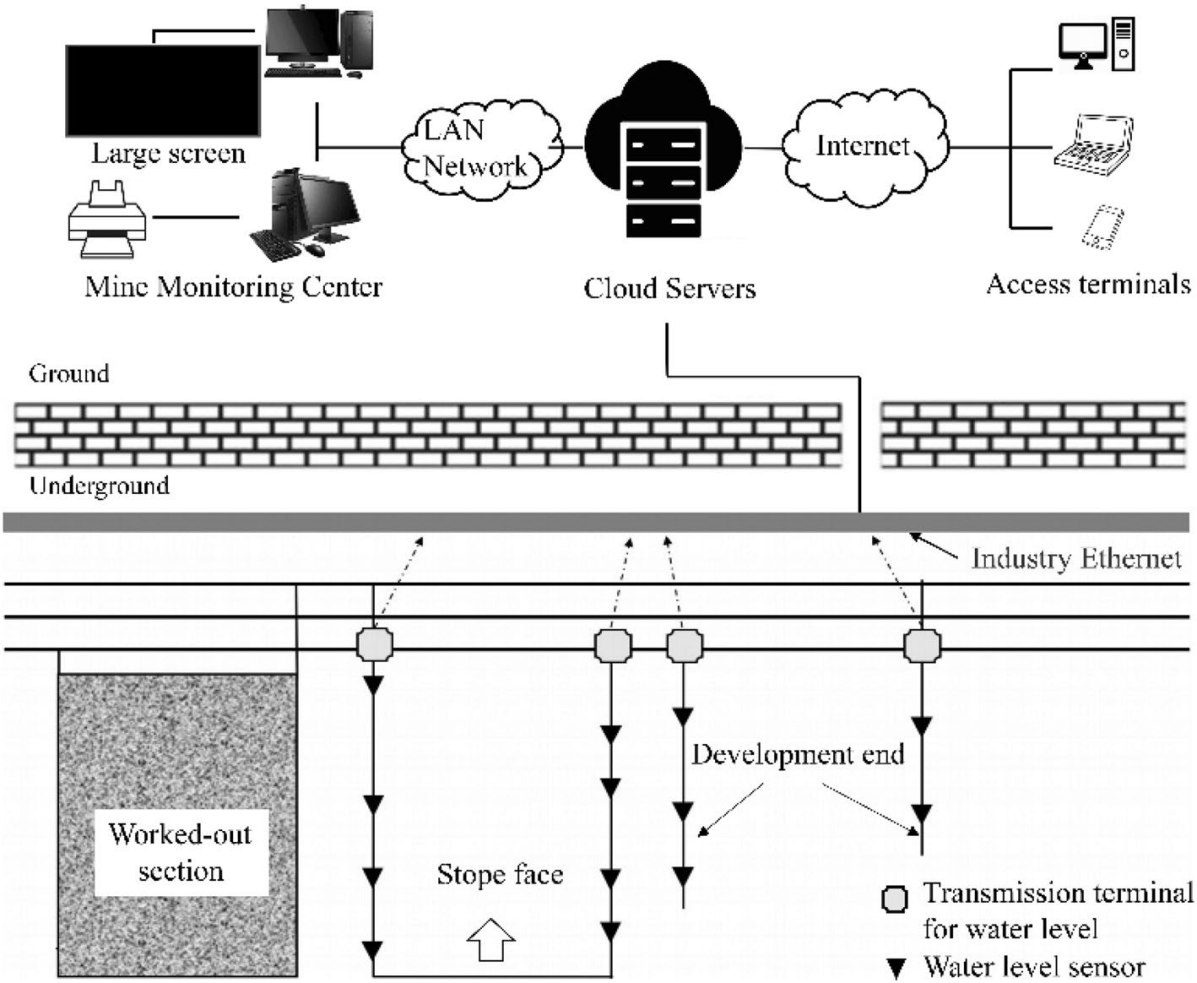
Schematic diagram of the proposed mine water inrush monitoring network.

The mine water inrush monitoring network proposed in this study was based on an underground network that monitored the depth of water flow in real time through the networked deployment of water level sensors at specific locations along a mine roadway (e.g., water sumps, drainage ditches, or low-lying areas of the roadway). This enabled changes in the amount of mine water inrush to be determined in a timely manner, as shown in Fig. 1.

The effectiveness of the monitoring network was largely determined by the installation location and number of water level sensors. Therefore, the selection of suitable locations for the water level sensors in the monitoring network to achieve a balance between cost and response time was a critical issue. A multi-constraint and multi-objective optimization layout method was therefore adopted to establish the layout of the monitoring network.

**Figure 2:**
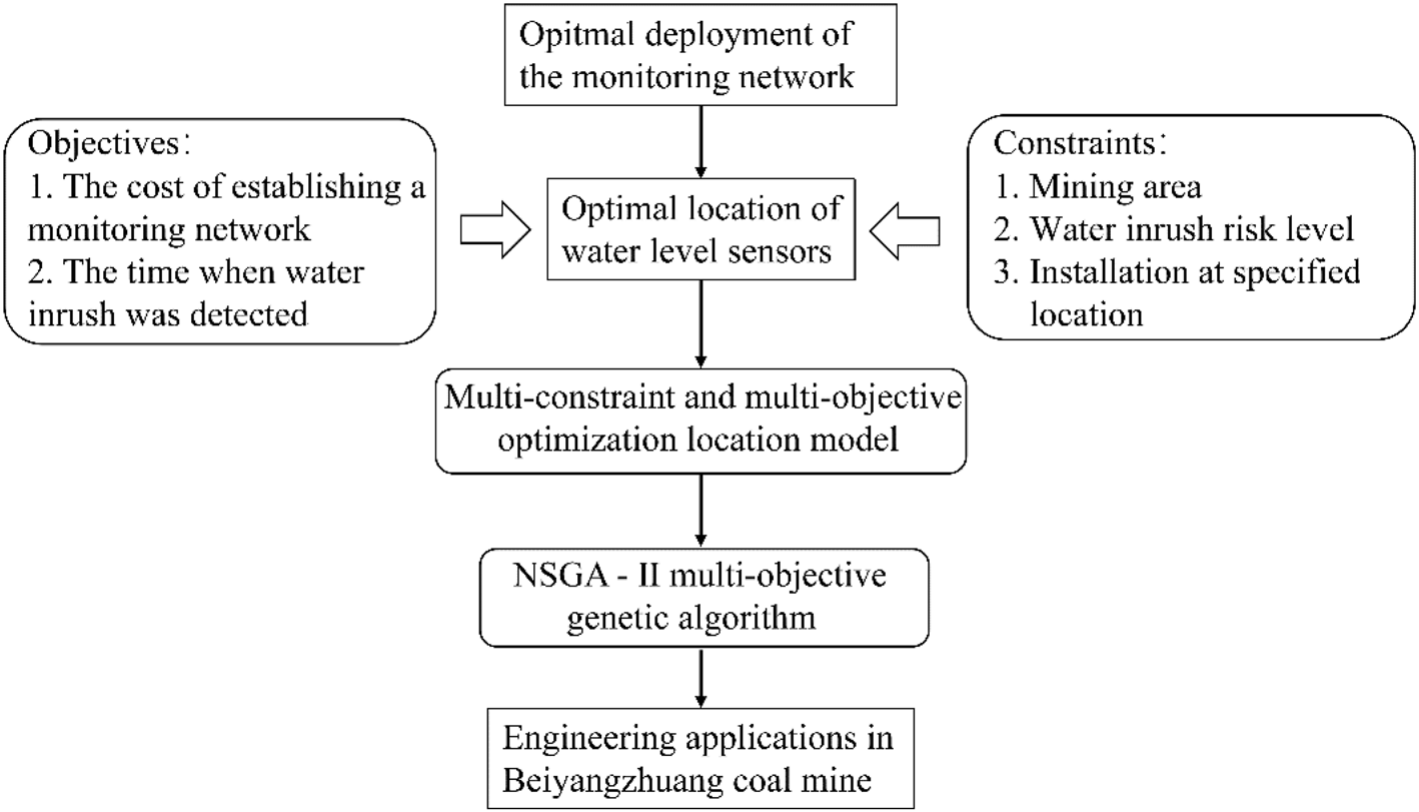
Flow chart of the multi-constraint and multi-objective optimization layout method.

With reference to Wu et al.^21^, an objective function and three multiple constraint conditions were incorporated to expand the problem of water level sensor locations into a multi-constraint and multi-objective optimization model, further enhancing the effectiveness and practicality of the monitoring network, as shown in Fig. 2.

### Mathematical model

#### Model parameters and decision variables

To fully explain the mathematical model, it is necessary to clarify the following concepts. The position in the roadway where a water level sensor can be installed is called a potential monitoring point, and the place where water inrush is simulated is called a water inrush point. Monitoring response time refers to the time that a water inrush is detected after its initial occurrence, represented by *T*. The spreading scope of a water inrush point refers to the roadway through which the water flow passes within the monitoring response time after the water inrush process is simulated at the water inrush point. The monitoring scope of a potential monitoring point refers to the area of roadway where the simulated water inrush can be monitored by the water level sensor installed at the potential monitoring point within the monitoring response time* T*. If a water inrush point is within the monitoring scope of a potential monitoring point, it means that the water inrush point can be monitored by the monitoring point. Therefore, within the monitoring response time *T*, the water inrush simulated at the water inrush point can be monitored by a water level sensor installed on the potential monitoring point. For the same potential monitoring point, the monitoring response time *T* is different, and the corresponding monitoring scope is also different.

Assuming that the number of water inrush points in a mine is *n*, each water inrush point corresponds to a fixed number *i*, starting from 0, then the set of water inrush points in the mine can be expressed as *N* = {0, 1, 2, 3, …, *i*}, *i* = n − 1. *N*_*i*_ represents the water inrush point numbered *i*, and *NT i* indicates the spreading scope of water inrush point *i* when the monitoring response time is *T*. If the number of potential monitoring points is *m*, each potential monitoring point corresponds to a fixed number *j*, starting from 0, then the set of potential monitoring points of the mine can be expressed as *M* = {0, 1, …, *m − *1}, *j* = *m − *1. *M*_*j*_ represents the potential monitoring point *j*, and *MT i*indicates the monitoring scope of the potential monitoring point* j* when the monitoring response time is *T*. No more than one water level sensor should be installed at the location of each potential monitoring point.

The decision variables are defined as follows:

1 $$ x_{j} = \left\{ {\begin{array}{*{20}l} {0,} \hfill \\ {1,} \hfill \\ \end{array} } \right. \quad \left( {j = 0, 1, 2, \ldots ,m - 1} \right) $$

When* x*_*j*_ is equal to 0, it means that no water level sensor is installed at the potential monitoring point *M*_*j*_; when *x*_*j*_ is equal to 1, it means that a water level sensor is installed at the potential monitoring point *M*_*j*_. The water inrush point *i* is monitored by at least one water level sensor within the monitoring response time *T*, i.e., at least one potential monitoring point in the spreading scope of the water inrush point *i* (*NT i*) must be installed with a water level sensor.

### Objective functions

#### Minimum total cost

The total cost of the mine water inrush monitoring network was uniformly expressed by the number of water level sensors, i.e., the fewer the water level sensors, the lower the total cost of the monitoring network. Combined with the decision variables, the objective function of minimum total cost was obtained as follows:

2 $$ f_{1} = min\mathop \sum \limits_{j} x_{j} \forall j \in M $$

This objective function was mainly used to optimize the location of water level sensors in the spatial domain.

#### Minimum average monitoring time

The average monitoring time refers to the average of the maximum monitoring time (*t*_max_) corresponding to all potential monitoring points where water level sensors were installed. The *t*_max_ of *M*_*j*_ is the latest time when water inrush within *MT j* was detected. Under the condition that the monitoring response time *T* is determined, the *t*_max_ of the potential monitoring point *M*_*j*_ can be calculated, and the value of *t*_max_ is less than or equal to the value of *T*. Combining the decision variables, the objective function of minimum average monitoring time was obtained as follows:

3 $$ f_{2} = min\left( {\frac{{\mathop \sum \nolimits_{j} x_{j} t_{max} }}{{\mathop \sum \nolimits_{j} x_{j} }}} \right) \forall j \in M $$

This objective function was mainly used to optimize the location of water level sensors in the time domain.

### Restraint conditions

1. No more than one water level sensor should be installed at each potential monitoring point:
  4 $$ x_{j} = \left( {0, 1} \right) \left( {j = 0, 1, 2, \ldots , m - 1} \right) $$
2. Within the monitoring response time T, each water inrush point should be monitored by at least one water level sensor:
  5 $$ \mathop \sum \limits_{{j \in N_{i}^{T} }} x_{j} \ge 1 \left( {i = 0, 1, 2, \ldots , n - 1} \right) $$
3. The monitoring response time T is the time requirement of monitoring water inrush when designing a monitoring network for a mine, and its unit is seconds:
  6 $$ T = t $$
4. Mining area
  The mining area refers to the area in the mine where excavation engineering construction is still ongoing, including slope faces and development ends. The roof and floor of coal seams in these areas are more seriously damaged, and mining cracks are more likely to penetrate the aquifer resulting in a water inrush hazard. Therefore, the optimized layout of a water inrush monitoring network in the mining area meets the requirements for preventing mine water disasters. The constraint conditions can be expressed by the following formula:
  7 $$ M_{area} = \left\{ {C_{1} , C_{2} , \ldots , C_{k} } \right\} $$
  *C*_*k*_ represents a mining area with sequence number *k,* and is also a set of some water inrush points.
5. Water inrush risk level
  Monitoring water inrush is critical in mining areas, especially in mining areas with a high water inrush risk level. Therefore, when selecting locations for the installation of water level sensors, the influence of the water inrush risk level should be considered. In locations with a high water inrush risk level there is a need for monitoring networks to rapidly determine the occurrence of water inrush.
  According to the hydrogeological conditions of a coal mine, a water inrush hazard zoning scheme can be applied using well-established water inrush hazard zonal-evaluation methods for the roof and floor of coal seams. For example, the three figures-dual prediction method has been applied to conduct a zonal evaluation of water hazards in a coal seam roof, and the vulnerability index method has been used to conduct a comprehensive zonal evaluation of water inrush risk for a confined aquifer in a coal seam floor^26,27^.
  Based on the location of the water inrush points in the mining area and the results of the water inrush risk zonal-evaluation of the mine, water inrush points were assigned different water inrush risk levels. The corresponding relationship between the water inrush risk level and the monitoring response time *T* was then established (Table 1). The corresponding relationship was dependent on the capacity of the drainage system in different mining areas and the time required for the monitoring network to detect a water inrush.

**Table 1:** Relationship between the water inrush risk level and monitoring response time (*T*).

Division of risk	Safe	Relatively safe	Transition region	Relatively dangerous	Dangerous
Risk level	1	2	3	4	5
*T (s)*	3600	3000	2100	900	600
  The judgment criterion used to determine the water inrush risk level was “care about high not about low”. For example, if there were three different water inrush risk levels (1, 3, and 4) in a development end, then the water inrush risk level of the development end was uniformly set to 4. According to Table 1, the corresponding monitoring network response time *T* should be 900 s. This constraint condition could be expressed by the following formula:
  8 $$ N_{i} \in I_{r} \left( {r \in 1, 2, 3, 4, 5} \right) $$
  where *r* refers to the water inrush risk level and *I*_*r*_ refers to the set of water inrush points for which the risk level is *r*.
6. Installation at specified locations
  The method for determining the optimal layout of the mine water inrush monitoring network was based on the premise that the water inrush per unit time was greater than the drainage capacity of the mining area or working face. Therefore, the location of the water level sensors was optimized to detect disastrous water inrush events, with a water volume greater than the drainage capacity. Generally, the probability of a disastrous water inrush in a mine is low. To ensure that the optimized location of the water level sensors considered the monitoring of the daily water inflow of a mine, a constraint condition for installing water level sensors at designated locations was proposed according to the location of the mine drainage system, water sumps, and monitoring points for daily water inflow. The constraint condition was expressed by the following formula:
  9 $$ x_{j} = 1, j \in D $$
  where *D* is the set of potential monitoring points at the specified location, and *j* is the identification number of a potential monitoring point.

### Algorithm design

The NSGA-II is an elitist non-dominated sorting genetic algorithm with an elite strategy and is one of the most popular multi-objective genetic algorithms^28^. It reduces the complexity of the NGSA and has the advantages of a fast running speed and good convergence of the solution set. The focus and difficulty of the NSGA-II genetic algorithm lies in the design of its genetic operation, which mainly includes the coding method, population size, selection, crossover, and mutation. The validity and feasibility of the solution should be considered when determining the optimal location of water level sensors. Using the NSGA-II multi-objective genetic algorithm to determine the optimal location of water level sensors, the legitimacy and feasibility of the solution was confirmed. The NSGA-II design steps are described in detail below.

1. Coding design
  A 0–1 coding scheme was adopted, in which 1 means a water level sensor was installed at a potential monitoring point, and 0 means that no water level sensor was installed. Assuming that the number of potential monitoring points was 10, Table 2 shows the chromosome of an individual monitoring point. The gene of this chromosome was 0010110100, which means that water level sensors were installed at the locations of the potential monitoring points numbered 2,4,5,7.

**Table 2:** Coding design of the NSGA-II algorithm.

Identification number	0	1	2	3	4	5	6	7	8	9
Gene	0	0	1	0	1	1	0	1	0	0
2. Initial population
  The initial population size was expressed by *P*, i.e., the number of individuals in the population. The value of *P* was set to 100.
3. Genetic operation
  Genetic operation consists of selection operation, crossover operation, and mutation operation. The selection operation adopted random sampling, the crossover operation adopted a two-point crossover, and the crossover probability was set to 0.8. The mutation operator of a binary chromosome was used, and the mutation probability was set to 0.1.
4. Termination conditions
  The running generation was selected as the termination condition of the algorithm, i.e., when the running generation was greater than the set value, the program stopped calculating. The operation generation was represented by *G*, and *G* was set to 60,000.

The NSGA-II multi-objective genetic algorithm was implemented using the Python programming language and “Geatpy” library function on the PyCharm compiler platform.

## Examples

Taking Beiyangzhuang coal mine in Hebei Province as an example, the proposed multi-constraint and multi-objective optimization layout method was used to establish a mine water inrush monitoring network. The mine was divided into four mining areas. The drainage capacities of mining areas I to IV were 800, 1000, 1200, and 2000 m^3^/h, respectively. The total drainage capacity of the central sump of the mine was 5080 m^3^/h.

### Data processing

#### Topological structure of the mine roadway

**Figure 3:**
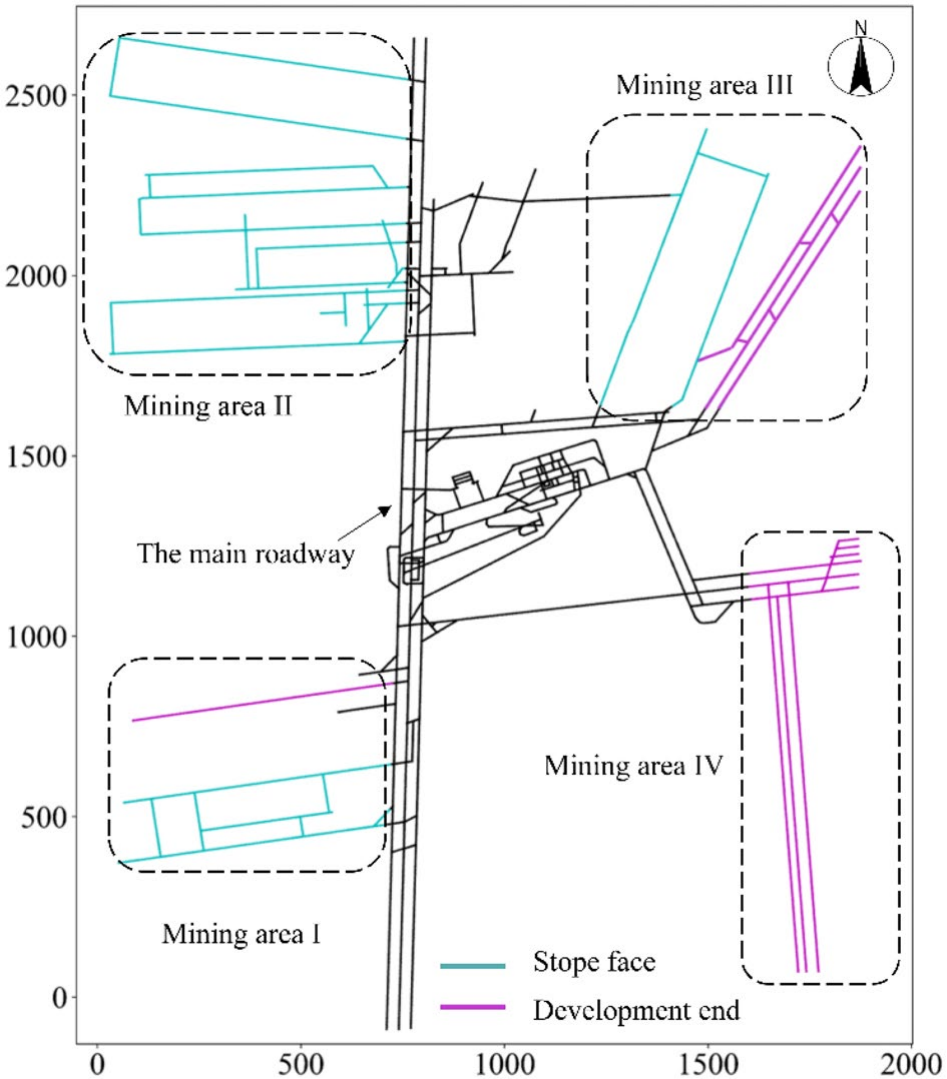
The mining area of the Beiyangzhuang coal mine.

The connectivity between roadways was established based on the mining engineering information for the mine. The number of traverse points along the mine roadway was 1658. On this basis, 8761 nodes were finally obtained using the numerical interpolation method^29^. The topological structure of the mine roadway was then established by these nodes and their connectivity (Fig. 3).

#### Mining area

A topological plan of the Beiyangzhuang coal mine was established based on the scope of the mining area, including slope faces and development ends, as shown in Fig. 3.

There were 3825 topological structure nodes in the mining area of the mine, which were also the water inrush points used to simulate the water inrush process.

#### Water inrush risk level

**Figure 4:**
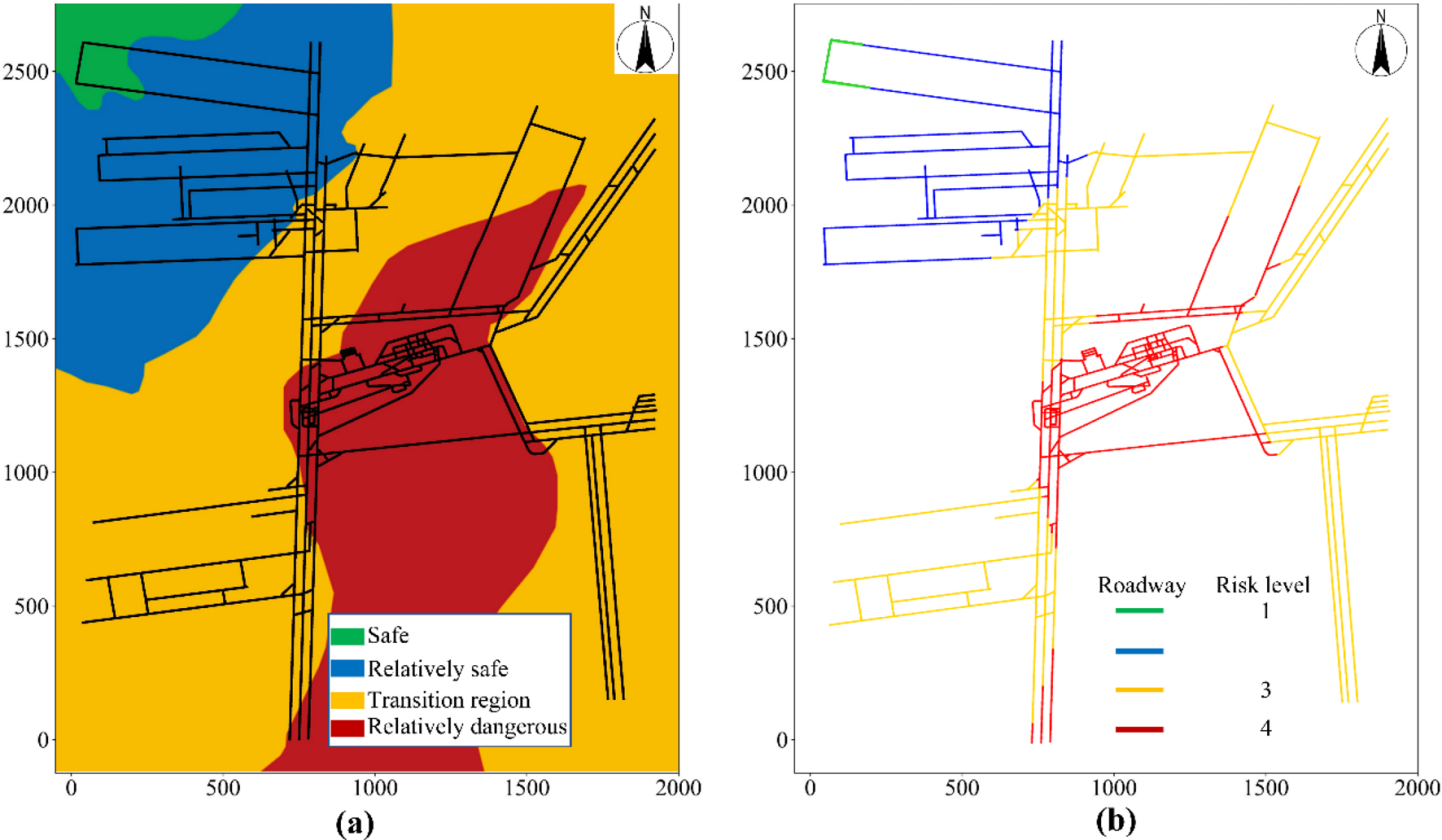
Water inrush risk level of mine roadways.

The vulnerability index method was used to conduct a comprehensive zonal evaluation of the water inrush risk for a confined aquifer in the coal seam floor of Beiyangzhuang coal mine (Fig. 4a). According to the comprehensive zonal evaluation of water inrush risk, the nodes on the mine roadway topology were divided into corresponding risk levels of water inrush, namely levels 1, 2, 3, and 4, which are shown in green, blue, yellow, and red, respectively, in Fig. 4b.

#### Installation at a specified location

**Figure 5:**
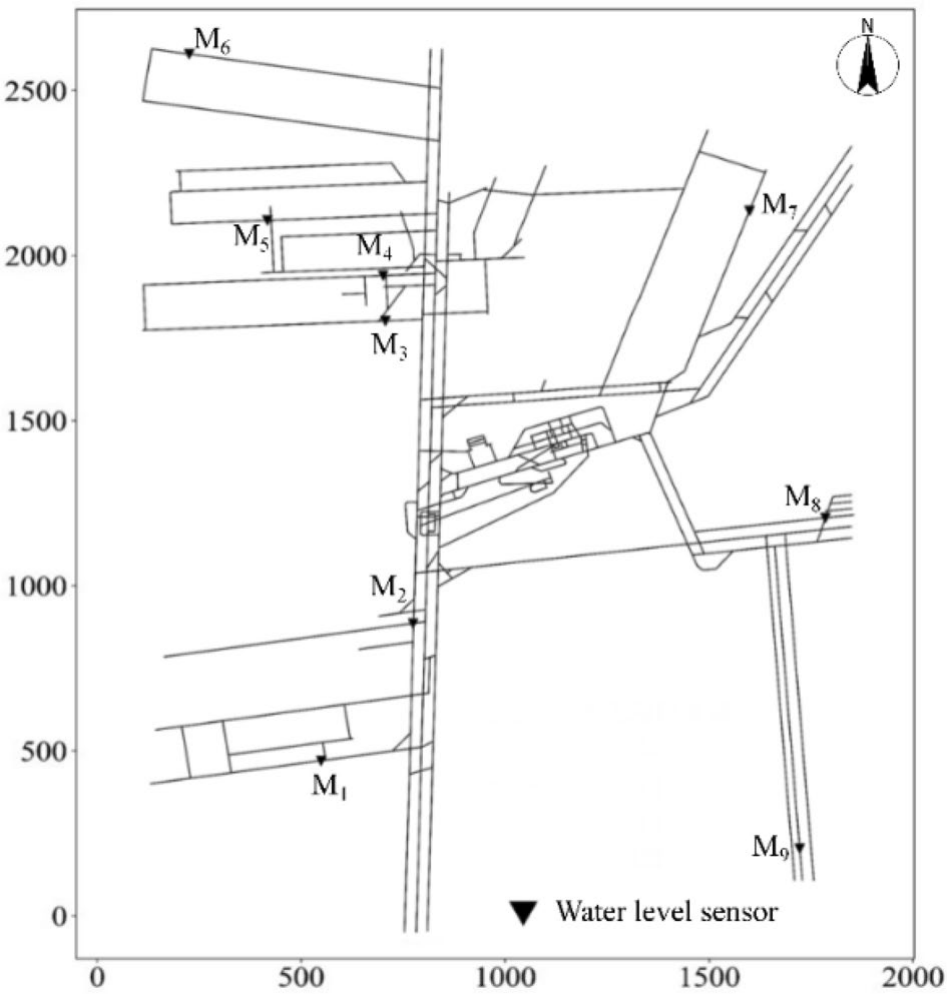
Specified positions for installing water level sensors.

Through an analysis of the mine drainage system, roadway elevation changes, and the location of monitoring points for daily water inflow in Beiyangzhuang coal mine, it was determined that water level sensors should be installed in the nine locations shown in Fig. 5.

#### The spreading scope of water inrush points

The water inrush process was simulated using the United States Environmental Protection Agency (USEPA) Storm Water Management Model (SWMM) after the topological structure of the mine roadway had been determined. The dynamic simulation of the water inrush spread process was constructed after building an SWMM model suitable for calculations along the mine roadway by analyzing the simulation method and parameter selection, and then setting reasonable boundary conditions^29^.

**Figure 6:**
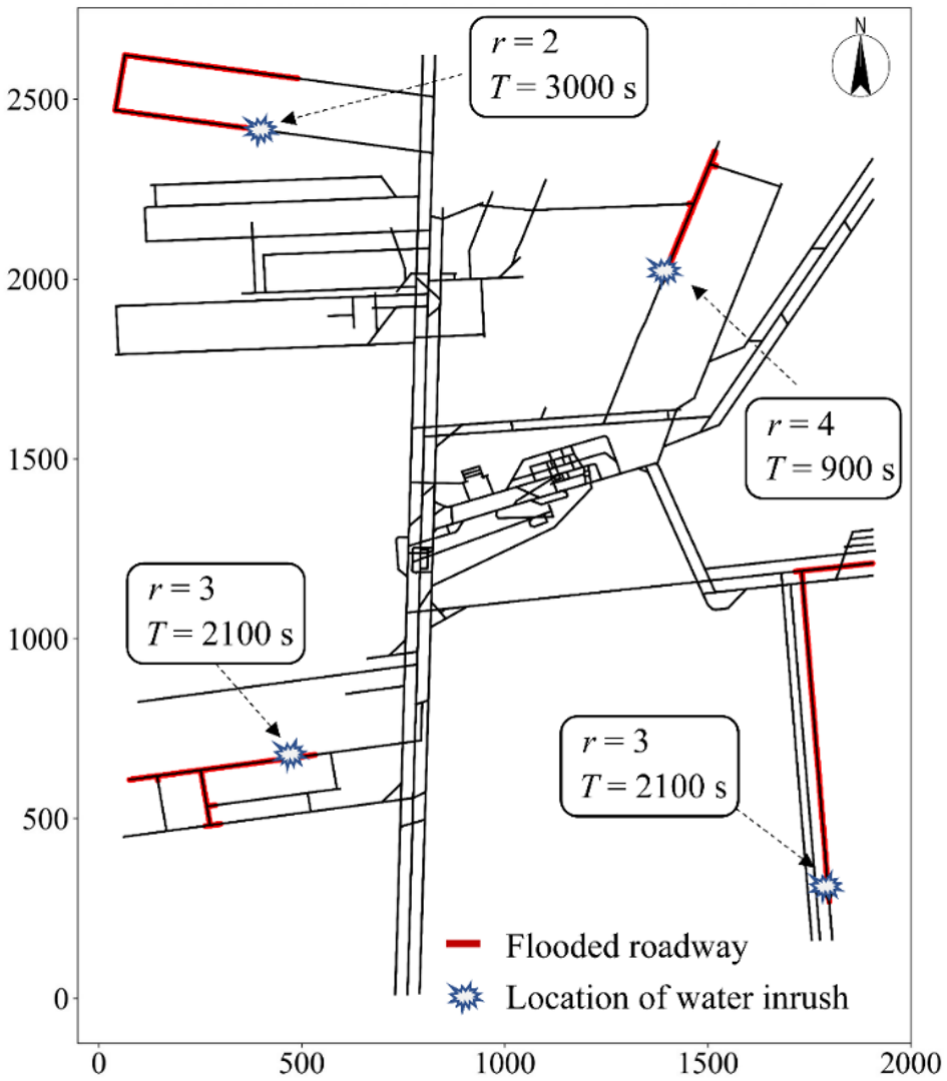
Spreading scopes of water inrush points.

Each topology node within the mining area was set as the water inrush point. The number of water inrush points was 3,825 in total, which was expressed by the following formula:* N* = {0, 1, 2, 3,…, *i*}, *i* = 3824. When using the SWMM software to simulate a disastrous water inrush, the magnitude of the water inrush was set according to the drainage capacity of the mining area where each water inrush point was located. The drainage capacities of mining areas I to IV in the Beiyangzhuang coal mine were 800, 1000, 1200, and 2000 m^3^/h, respectively. The spreading scope of each water inrush point within the monitoring response time *T* could be obtained according to the data for the simulated water inrush. At different monitoring response times *T*, the spreading scope of the water inrush point also differed. It can be seen from Table 1 that the corresponding monitoring response time *T* could be obtained by the water inrush risk level at the location of the water inrush point. Figure 6 shows the spreading scope (the thick red lines) of four randomly selected water inrush points.

#### The monitoring scope of potential monitoring points

**Figure 7:**
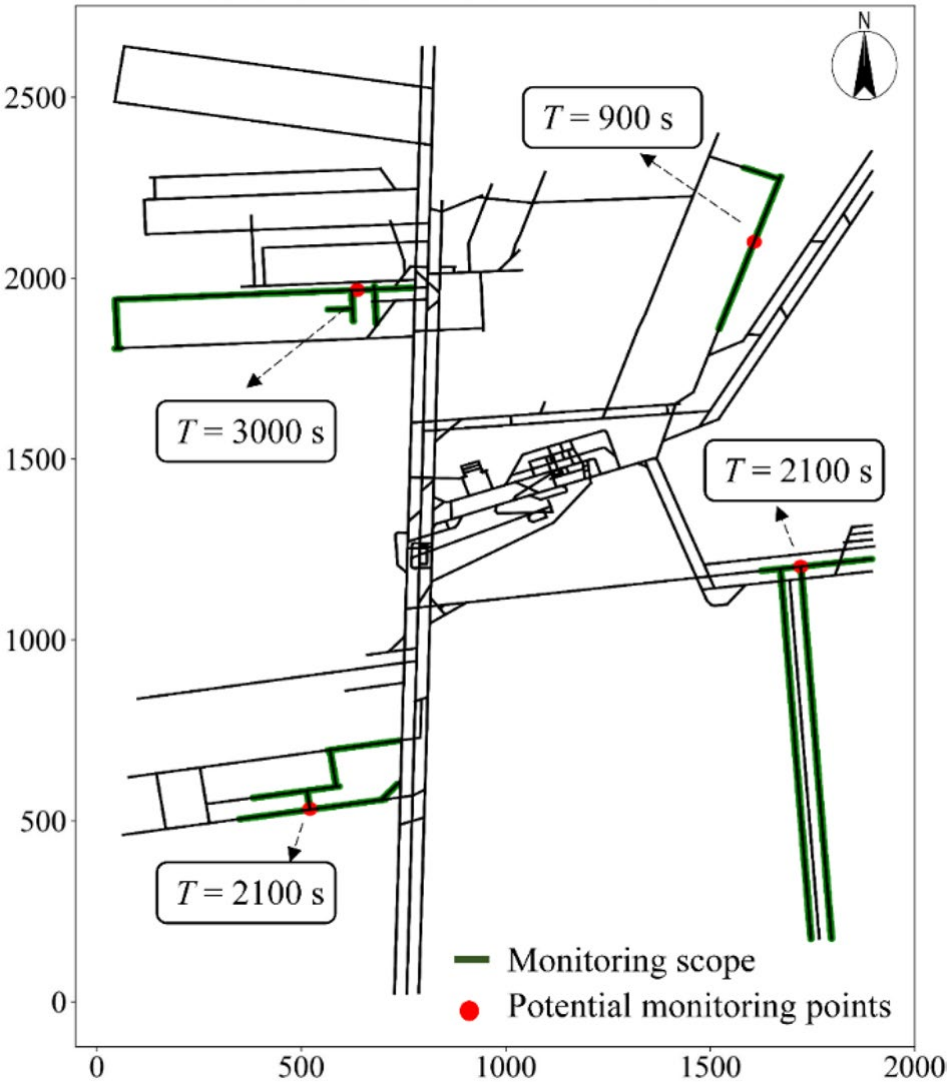
Monitoring scopes of the potential monitoring points.

From the spreading scope of the water inrush points, the potential monitoring points and their monitoring scopes could be obtained. They were expressed in the following forms:

10 $$M= \left\{{M}_{0}^{T}, {M}_{1}^{T}, {M}_{1}^{T}, \dots , {M}_{1}^{T}\right\}, j=(0, 1, 2, 3, \dots , m-1)$$

where *m* is number of potential monitoring points (*m* = 3886). With different monitoring response times *T*, the monitoring scope of the potential monitoring points differed. Figure 7 shows the four potential monitoring points that were randomly selected and their corresponding monitoring scopes (the thick green lines).

## Results and discussion

**Figure 8:**
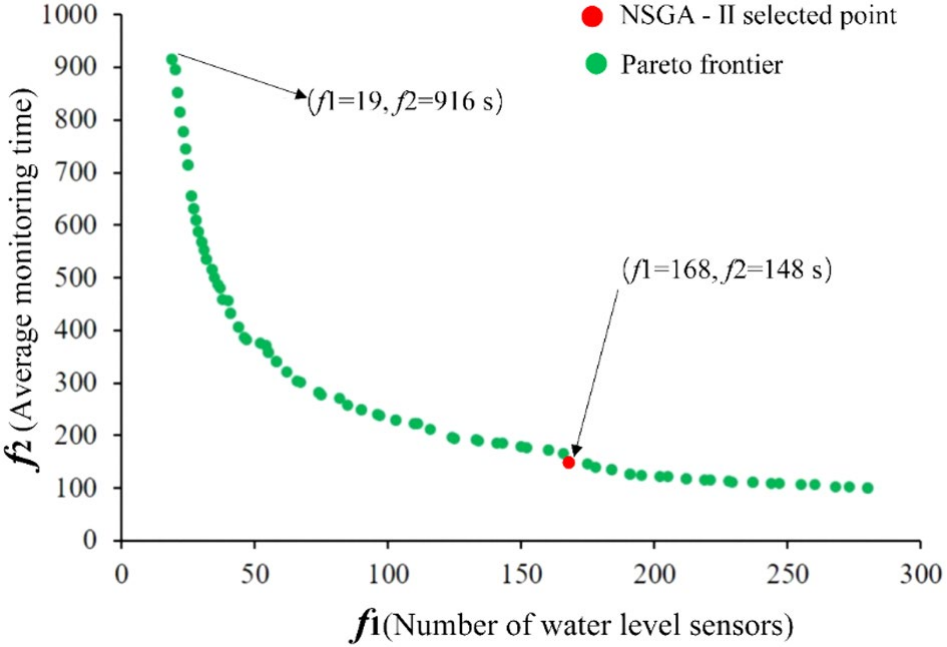
The Pareto solution of the multi-constraint and multi-objective optimization layout method.

Using the Python programming language to run the NSGA-II multi-objective genetic algorithm on the PyCharm compiler platform, the Pareto optimal solution and Pareto front were obtained (Fig. 8). It can be seen from Fig. 8 that the NSGA-II genetic algorithm successfully solved the multi-constraint multi-objective location model, and the algorithm had a strong convergence.

**Figure 9:**
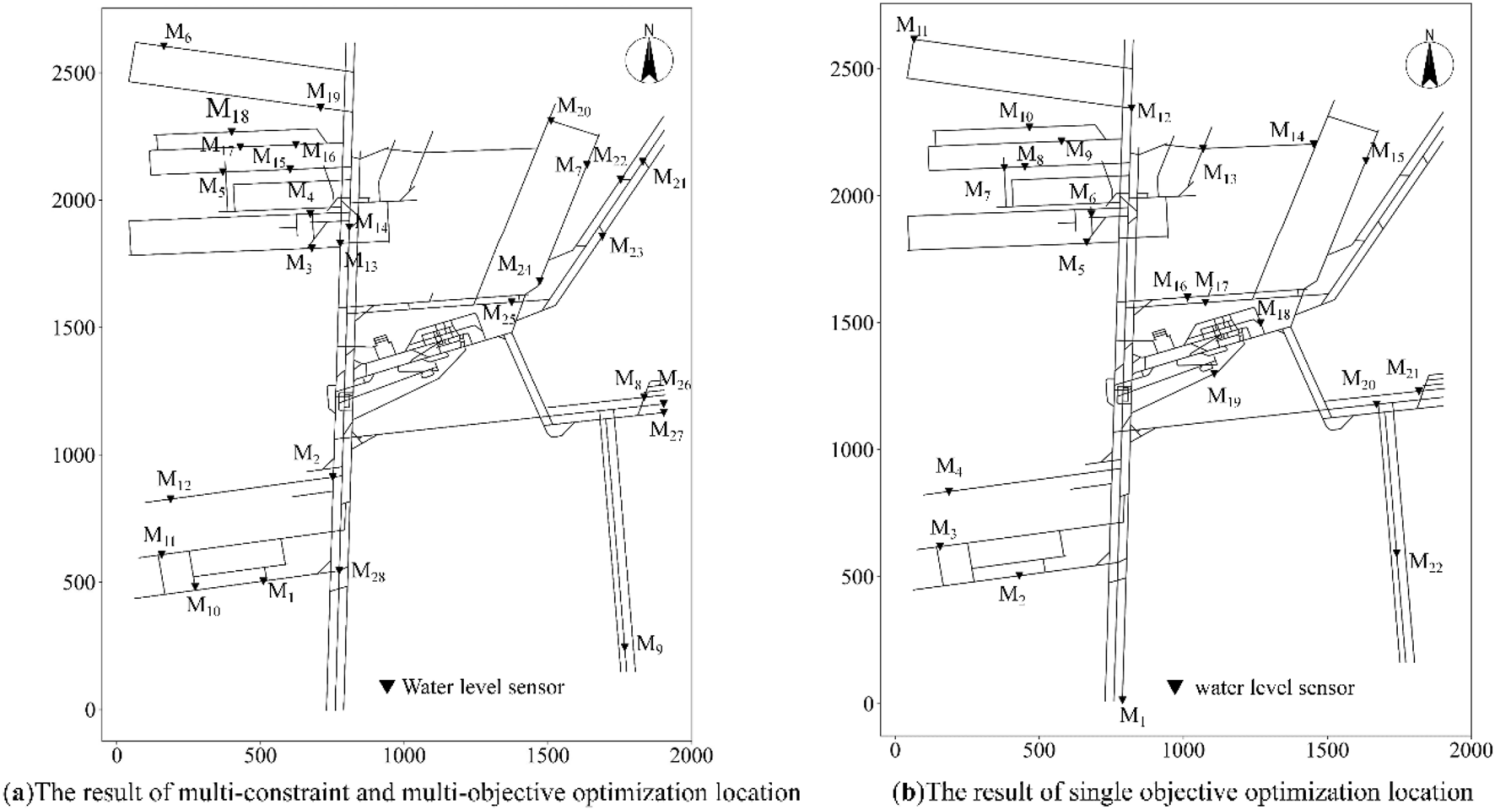
Deployment scheme of a monitoring network composed of 28 water level sensors.

The Pareto frontier shown in Fig. 8 was composed of feasible Pareto solutions. The results balanced the cost and time objectives and provided a variety of different deployment schemes. The optimal solution obtained by the NSGA-II multi-objective genetic algorithm was (*f*_1_ = 168, *f*_2_ = 148 s). Therefore, when 168 sensors were installed, the average time required for a water inrush to be detected in the mining area could be controlled to within 148 s, ensuring that construction costs and mine safety (The time when water inrush was monitored) reach a cost-effective balance. Due to the increasing scale of mining, the construction cost of a monitoring network is high, and therefore coal mines tend to choose solutions with lower construction costs. For example, if the decision is based on the cost, the solution (*f*_1_ = 19, *f*_2_ = 916 s) with the lowest cost can be selected, i.e., a mine water inrush monitoring network composed of 28 water level sensors, including nine specified locations (Fig. 9a).

Due to the additional constraints on the mining area and water inrush risk level, the deployment scheme in Fig. 9a could not only monitor water levels leading to disastrous water inrush accidents in the mining area of the Beiyangzhuang coal mine, but it also shortened the monitoring response time *T* at the high water inrush risk level. A comparison with Fig. 9b shows that the locations of the monitoring points were concentrated in the mining area in Fig. 9a, and the number of monitoring points in areas with a high risk level of water inrush also increased accordingly. This concurred with the technical requirements for water inrush prevention and control in mines. The average response time required to detect a catastrophic water inrush by the monitoring network was within 916 s after deploying only 28 water level sensors in the network. Due to the considerations made in the location of the nine daily water inflow monitoring points (i.e., the constraints of installation at the specified locations), this method could monitor daily water levels in coal mines without a water inrush.

**Figure 10:**
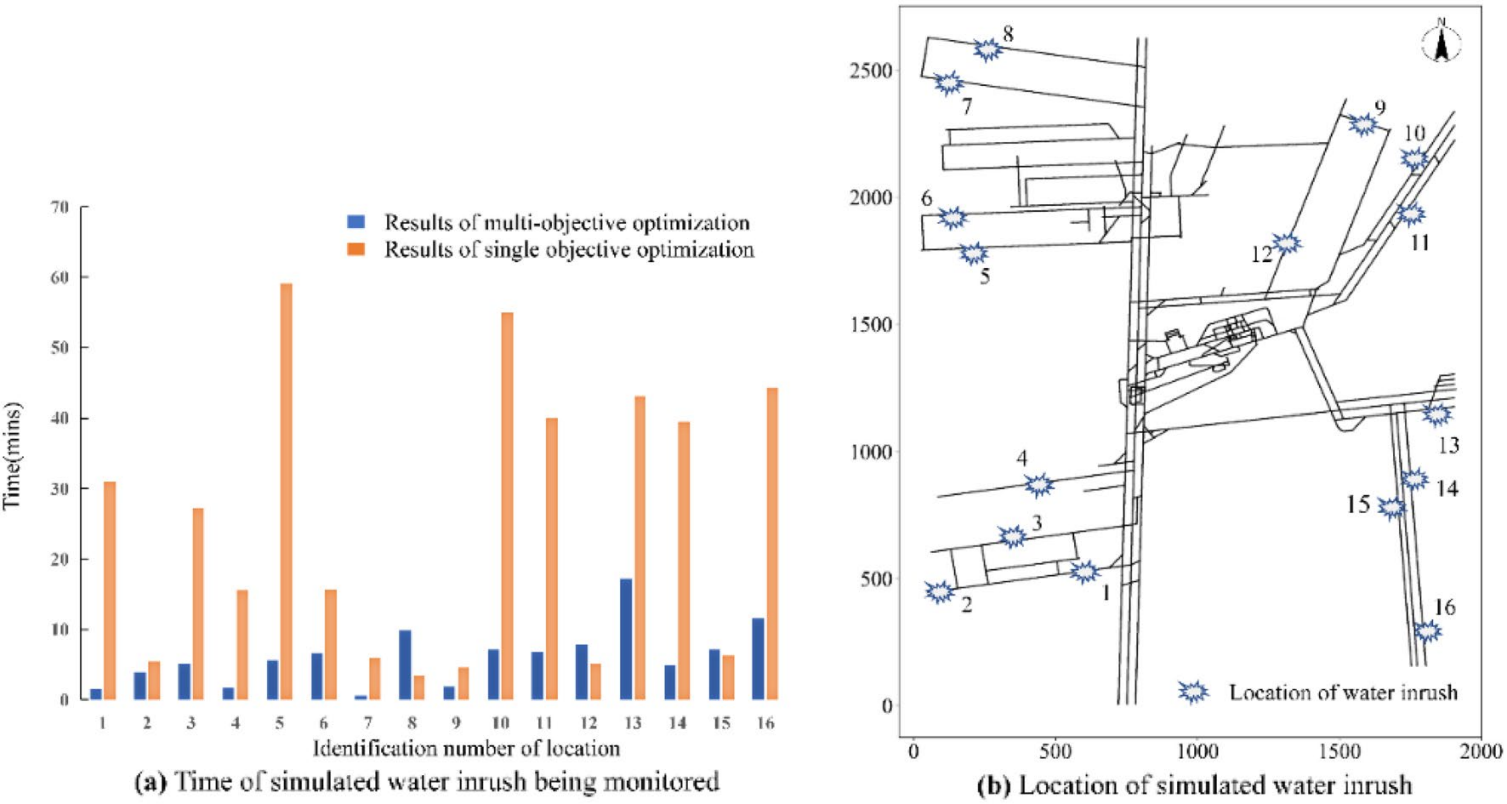
Verification of the monitoring response times of the deployment schemes.

The solution obtained by the multi-constraint and multi-objective optimization method can ensure a more reasonable selection of the installation position of water level sensors from the perspective of monitoring time. Sixteen locations (Fig. 10b) were randomly selected within the mining area to simulate the process of water inrush. Then, the occurrence of water inrush was monitored using the two deployment schemes shown in Fig. 9. The solution obtained by the multi-constraint and multi-objective optimization method detected the occurrence of water inrush over a shorter monitoring time (Fig. 10a).

Compared with the single objective optimization results, even if the layout scheme with the least cost was selected, the multi-constraint multi-objective optimization method made the best choice for the location of monitoring points in terms of monitoring time, mining area, and risk level of water inrush.

### Validation of results

Under the constraint conditions, i.e., mining area, water inrush risk level, and installation at specified locations, whether the scheme could achieve the monitoring effect using the multi-constraint and multi-objective optimization layout method was verified in terms of the following two aspects.

### Monitoring scope

To verify whether the water inrush in the mining area of the Beiyangzhuang coal mine could be monitored, the monitoring scope of each water level sensor was expressed in the form of its minimum circumscribed polygon. If the combined monitoring scope of the 28 water level sensors covered the mining area, it was confirmed that the water inrush in the mining area of the mine could be monitored by the suggested deployment scheme.

**Figure 11:**
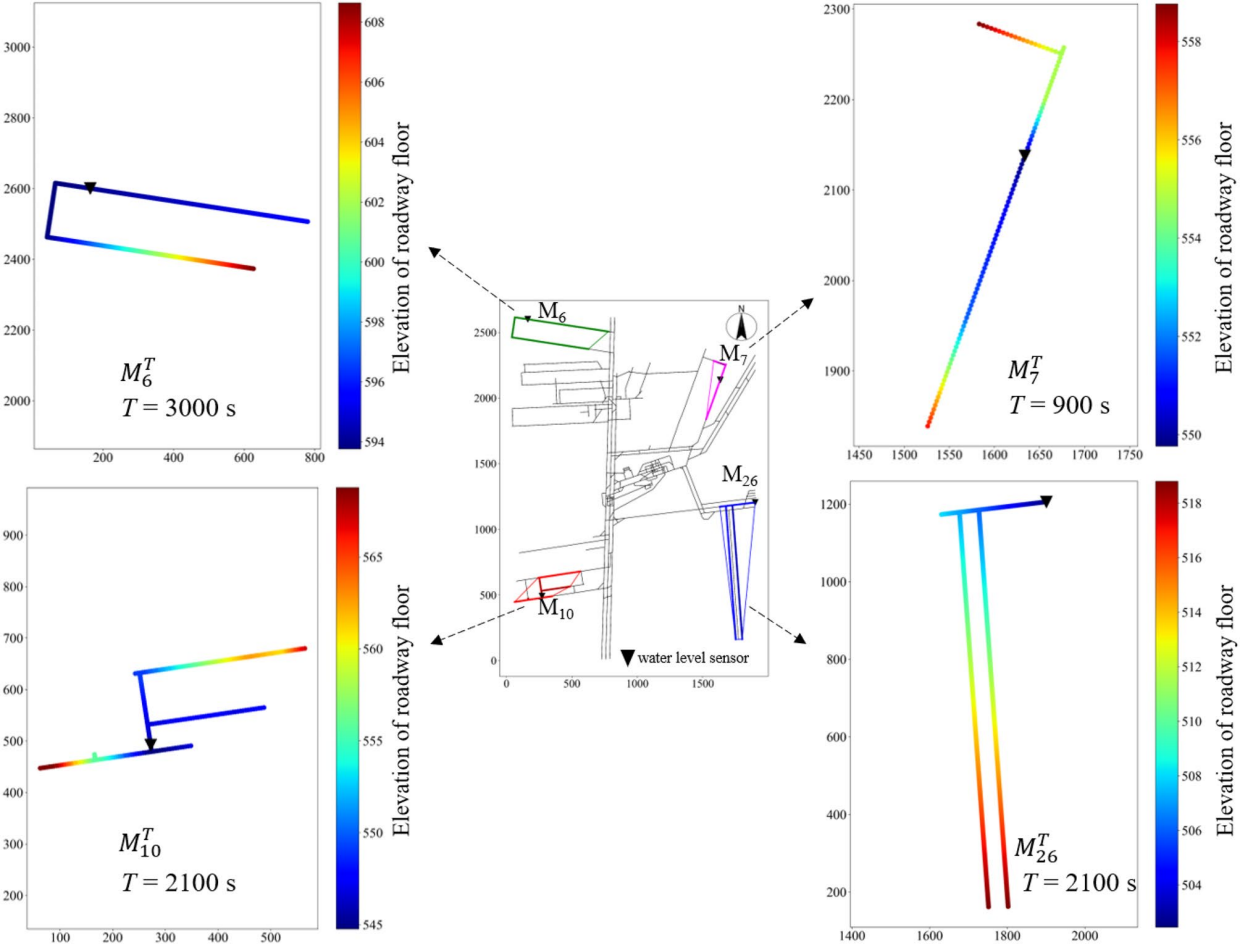
Monitoring scope of water level sensors (*M*_6_, *M*_7_, *M*_10_, *M*_26_).

Figure 11 shows the monitoring scope of four randomly selected monitoring points (*M*_6_, *M*_7_,* M*_10_, *M*_26_) from among the 28 water level sensors and their minimum circumscribed polygons. To display the relationship between the installation position of water level sensors and the roadway elevation more intuitively, a gradual change in color was used to represent the roadway elevation change within each monitoring scope.

**Figure 12:**
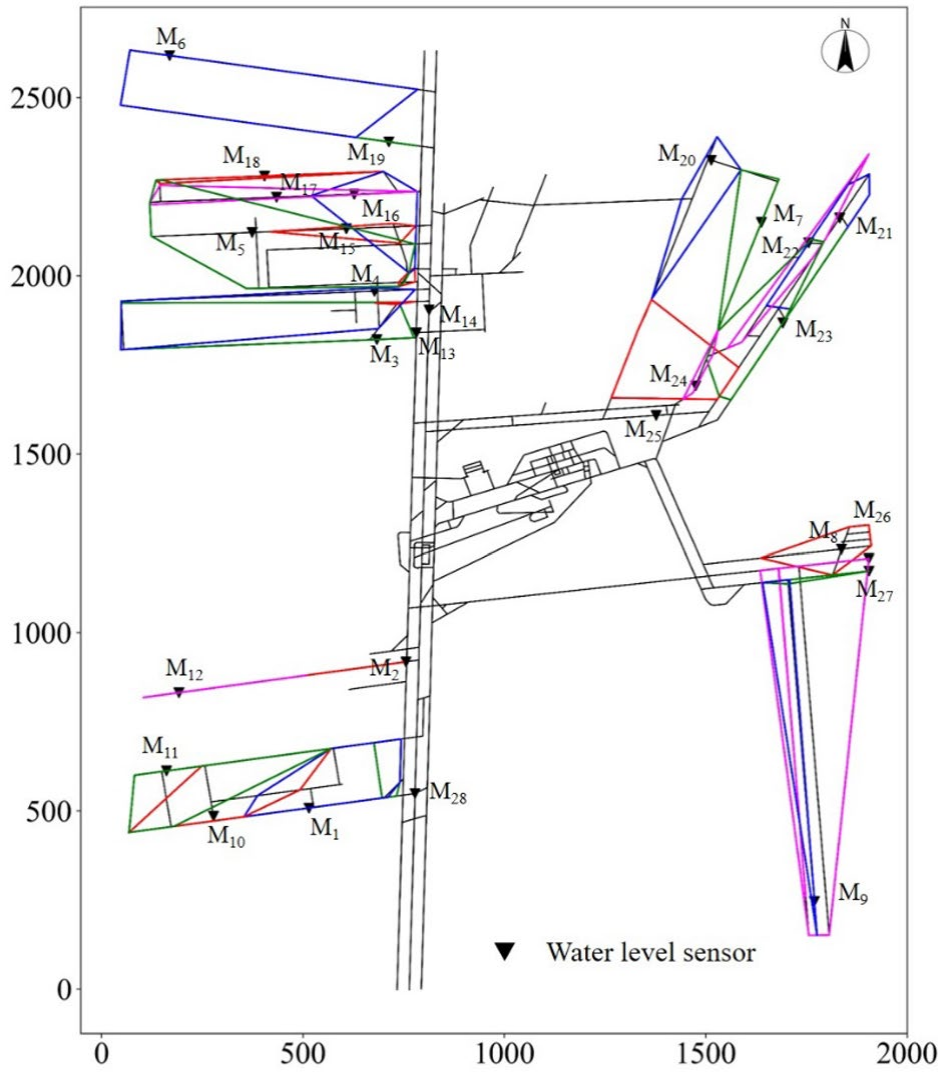
Verification of the monitoring scope of the sensor deployment scheme.

It can be seen from Fig. 12 that the monitoring scope of 28 water level sensors completely covered the mining area, which proved that the deployment scheme could effectively monitor the water inrush in the mining area of the mine.

### Monitoring response time

**Figure 13:**
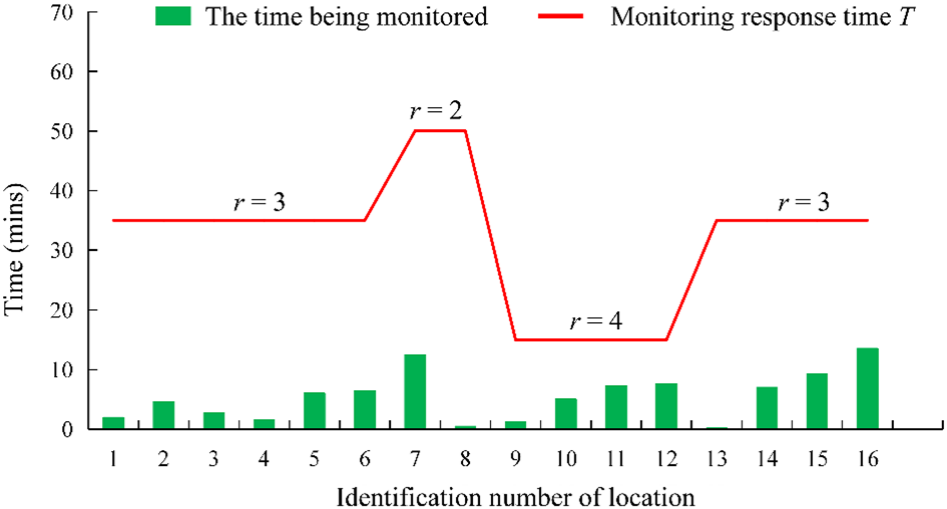
Verification of the monitoring response time of the sensor deployment scheme.

To verify whether the monitoring response time *T* of the deployment scheme was shortened with an increase in the water inrush risk level, 16 locations (Fig. 10b) were randomly selected in the mining area to simulate the water inrush process and the time when the water inrush was detected was recorded. It can be seen from Fig. 13 that the monitoring network response time *T* decreased with an increase in the water inrush risk level. The time taken to detect the simulated water inrush was less than the corresponding monitoring response time *T*. Therefore, the deployment scheme could realize the monitoring of water inrush in the mining area of the mine within the monitoring response time *T*.

## Conclusions

Water inrush disasters in coal mines are declining in frequency due to the continuous improvements in prevention and treatment technologies, but disasters that cause casualties can still occur periodically. In this study, an underground monitoring network for disastrous water inrush accidents in coal mines was proposed, and a multi-constraint and multi-objective optimal layout method was then established. The method optimized the monitoring network in the spatial and temporal dimensions, which reduced the cost and improved the monitoring response time.

1. A water inrush monitoring network was established by deploying a series of water level sensors in mine tunnels. Two objective functions were constructed: minimum total cost and minimum average monitoring time. A multi-objective optimization model for water level sensors was established, which reduced the cost while improving the response time of the monitoring network.
2. Three practical constraints of mining area, water inrush risk level, and installation at specified locations were established and quantified, and then a multi-constraint and multi-objective optimization model was established. The two constraints of mining area and water inrush risk level were used to concentrate the location of monitoring points in areas with mining activities and areas with a high water inrush risk on the roof and floor of coal seams, which concurred with the technical requirements for water inrush prevention and control in mines. The constraint condition of installation at specified locations enabled the monitoring network to undertake the task of monitoring the daily water inflow of the mine without water inrush, expanding the scope of use of the monitoring network.
3. The NSGA-II multi-objective genetic algorithm was designed with Python programming language to solve the model, and a Pareto front with a good convergence was obtained. The proposed method was used to realize the optimal layout of a water inrush monitoring network in Beiyangzhuang coal mine, Hebei Province. The average response time taken for a catastrophic water inrush to be detected in the mining area was within 916 s with the networked deployment of just 28 water level sensors in the mine roadway. Under the water inrush risk levels of 2, 3, and 4, the response time for simulated water inrush accidents was less than 3000, 2100, and 900 s, respectively. Due to the constraints of the installation at specified locations (nine daily water inflow monitoring points), the monitoring network could also be used for monitoring daily water inflows in the mine.

## Data Availability

The datasets used and analyzed during the current study available from the corresponding author on reasonable request.

## List of symbols

*i*: The serial number of water inrush points
*j*: The serial number of potential monitoring points
*n*: The number of water inrush points
*k*: The number of mining areas
*N*: The serial number of water inrush points
*N*_*i*_: The water inrush point numbered *i*
*T*: The monitoring response time
$N_{i}^{T}$: The spreading scope of water inrush point *i* under *T*
*m*: The number of potential monitoring points
$M_{j}^{T}$: The monitoring scope of potential monitoring point *j* under *T*
*C*_*k*_: The mining area with sequence number *k*
*r*: The risk level of water inrush
*I*_*r*_: The set of water inrush points that the risk level is *r*
NSGA-II: The non-dominated sorting genetic algorithm II
